# From plant disease to protective agent: tea blister blight aqueous extract restore copper-induced oxidative and inflammatory damage in zebrafish model

**DOI:** 10.3389/fnut.2026.1916713

**Published:** 2026-08-11

**Authors:** Jiaqi Liu, Nguyen Huy Hoang, Yuqing Ma, Song Tang, Haiyang Liu, Jiayan Long, Jiajia Zhang, Ren Mu, Xiaolu Zhou, Caibi Zhou

**Affiliations:** 1 Key Laboratory of National Forestry and Grassland Administration on Biodiversity Conservation in Southwest China, Yunnan Academy of Biodiversity, Key Laboratory of Forest Disaster Warning and Control in Yunnan Province, Ancient Tea Tree Research Center, College of Forestry, Southwest Forestry University, Kunming, China; 2 College of Biological Science and Agriculture, Qiannan Normal University for Nationalities, Duyun, China; 3 School of Pharmaceutical Sciences, Guizhou Medical University, Guiyang, China; 4 School of Crop Production Technology, Institute of Agricultural Technology, Suranaree University of Technology, Nakhon Ratchasima, Thailand; 5 Tea Research Institute, Yunnan Academy of Agricultural Sciences, Kunming, China

**Keywords:** antioxidant, copper sulfate, inflammation, tea blister blight, zebrafish embryo

## Abstract

**Background:**

Oxidative stress and inflammation play key pathological mechanisms involved in the development of chronic diseases. Although tea blister blight reduces commercial tea quality, pathogen-induced stress may enhance the accumulation of bioactive phenolics and flavonoids, making infected leaves a potential resource for the food-medicine homology framework. This study aimed to evaluate the protective effects of a tea blister blight aqueous extract (TBB) against copper sulfate (CuSO_4_)-induced oxidative and inflammatory stress in zebrafish (*Danio rerio*) embryos.

**Methods:**

Zebrafish embryos were exposed to 3 μM CuSO_4_ and co-treated with TBB at concentrations of 0.3–0.9 mg/mL. Developmental toxicity was evaluated based on survival, hatching, and morphological outcomes. Oxidative stress was assessed by measuring malondialdehyde (MDA) levels and antioxidant enzyme activities, including superoxide dismutase (SOD) and catalase (CAT). The expression of genes related to antioxidant defense, inflammation, and apoptosis was also analyzed.

**Results:**

TBB treatment significantly increased embryo survival by 12.74–15.81% and the hatching rate by 46.12–51.38% than the CuSO_4_-exposed group. TBB also reduced CuSO_4_-induced developmental toxicity by increasing body length by 15.38–16.35% and improving the sinus venosus–bulbus arteriosus distance by 10.40–14.80%. In addition, TBB significantly mitigated oxidative stress by reducing MDA levels by 16.74–25.61% and enhancing SOD and CAT activities by 31.29–72.35% and 69.66–92.37%, respectively. Gene expression analysis showed that TBB upregulated antioxidant-related genes (*Mn-sod*, *CAT*, and *Gpx1a*), suppressed proinflammatory genes (*TNF-α*, *NF–κB*, *NLRP3*, and *IL-1β*), and downregulated the pro-apoptotic gene *bax*, while enhancing anti-inflammatory (*IL-10*) and anti-apoptotic (*Bcl-2*) expression.

**Conclusion:**

These findings demonstrate that TBB provides multilevel biological protection through antioxidant, anti-inflammatory, and anti-apoptotic mechanisms. Importantly, this study provides a new perspective by demonstrating that tea leaves infected with tea blister blight disease, rather than being discarded or managed solely through fungicide application, can be valorized as a bioactive-enriched resource for applications in functional nutrition and health-promoting products.

## 1 Introduction

Oxidative stress is an imbalance between the generation of reactive oxygen and nitrogen species (ROS/RNS) and the body’s antioxidant defense system. When oxidant production exceeds antioxidant capacity, excessive ROS, such as superoxide anions, hydrogen peroxide, and hydroxyl radicals react with cellular components including lipids, proteins, and nucleic acids, thereby disrupting redox homeostasis and leading to oxidative damage (1, 2). Long-term oxidative stress causes structural and functional impairment of cells through lipid peroxidation, protein denaturation, and DNA mutations, which can lead to apoptosis or necrosis. It has been implicated in more than 50 pathological conditions, including diabetes, hypertension, cancer, cardiovascular, and neurodegenerative diseases (3, 4). Inflammation is a complex biological defense mechanism triggered by harmful stimuli such as pathogens, toxins, or tissue injury, involving immune cells, cytokines, and molecular mediators that act to eliminate the injurious stimulus and restore tissue homeostasis (5). Long-term oxidative stress enhances inflammatory signaling through *NF–κB*, *MAPK*, and cytokine cascades, whereas inflammation itself promotes overproduction of ROS, forming a self-perpetuating cycle that amplifies cellular injury and tissue dysfunction (6, 7). Apoptosis, or programmed cell death, is a regulated process that maintains cellular homeostasis by removing damaged or aged cells through intrinsic (mitochondrial) and extrinsic (death receptor-mediated) pathways, primarily mediated by caspases (8). These three processes are intimately interconnected; oxidative stress and inflammation can trigger apoptosis, whereas dysregulated apoptosis can, in turn, release proinflammatory signals and reactive molecules, ultimately contributing to tissue degeneration and the progression of various chronic diseases (8, 9).

The zebrafish (*Danio rerio*) has become a versatile vertebrate *in vivo* model widely used to screen drugs and natural compounds for diverse diseases, including neurological, cardiovascular, metabolic, inflammatory, and oxidative stress-related disorders, because of its genetic similarity to humans, transparent embryos, and suitability for high-throughput assays (10–12). In particular, zebrafish models have proven valuable in evaluating antioxidant activity, enabling real-time observation of ROS generation, antioxidant enzyme expression, and redox balance restoration, thus providing a rapid and reliable system for identifying compounds with protective effects against oxidative damage (13, 14). Copper sulfate (CuSO_4_) is widely used as a model compound to induce oxidative stress in animal models because it readily releases Cu^2+^ ions that catalyze the generation of reactive oxygen and nitrogen species (ROS/RNS), leading to lipid peroxidation, protein oxidation, and depletion of antioxidant enzymes, such as SOD, CAT, and GSH (15, 16). The excessive ROS induced by CuSO_4_ activates pro-inflammatory signaling pathways, including *NF–κB* and cytokine release (*TNF-α*, *IL-6*), and triggers mitochondrial dysfunction, which together promote apoptosis through caspase activation and neuronal or cellular degeneration, highlighting the interconnected mechanisms of oxidative stress, inflammation, and apoptosis in CuSO_4_-induced toxicity (15–17). In recent years, the CuSO_4_-induced zebrafish model has been used to evaluate the antioxidant and anti-inflammatory activities of various bioactive compounds (acacetin, squalene, *β*-sitosterol, 2′-hydroxychalcone) and also herbal extracts (*Chenopodium album*, *Clerodendrum cyrtophyllum*, *Talisia esculenta*). These compounds and extracts significantly attenuated CuSO_4_-induced oxidative stress, reduced ROS accumulation, suppressed proinflammatory cytokine expression, and inhibited macrophage or neutrophil migration (18–24).

Tea (*Camellia sinensis*) is the world’s most consumed functional beverage after water and is rich in bioactive compounds such as polyphenols, catechins, and amino acids, which have been linked to the prevention and management of non-communicable chronic diseases including cardiovascular diseases, diabetes, obesity, and cancer through antioxidant, anti-inflammatory, and metabolic regulation mechanisms (25–27). Blister blight caused by *Exobasidium vexans* Massee is one of the most destructive foliar diseases of tea, infecting tender leaves, buds, and young stems across nearly all tea-growing regions of Asia (28). The disease can cause yield losses of 33–50% and significantly decrease the biochemical composition and sensory quality of tea by decreasing the levels of polyphenols, catechins, and caffeine while altering flavor characteristics. Its recurring epidemics under humid and cool conditions pose a persistent threat to the global tea industry, affecting both productivity and market value (29). Infection triggers a metabolic shift in the flavonoid pathway, leading to the isomerization of proanthocyanidins from 2,3-trans (catechin, gallocatechin) to 2,3-cis (epicatechin, epigallocatechin) forms and increased gallate esterification of catechin subunits (30). These changes are associated with elevated levels of defense-related phenolics, anthocyanins, and proanthocyanidins, while key quality components such as EGCG, sugars, and chlorophyll decline, reducing the sensory quality of tea (31, 32). Previous research has shown that *E. vexans* infection causes extensive alterations in tea leaf metabolites, notably increasing flavonoids, phenolic acids, alkaloids, and terpenoids, while enriching the flavone, flavonol, and phenylpropanoid pathways associated with enhanced antioxidant and antimicrobial activities (33). Although plant diseases are generally associated with negative impacts on crop yield and quality, some endophytic or pathogenic fungi can stimulate the accumulation of valuable secondary metabolites in host plants (34, 35). These interactions may enhance the production of bioactive compounds in herbs, including flavonoids, alkaloids, and terpenoids with potential pharmaceutical or nutraceutical applications if effectively utilized (36, 37). The modern food-medicine homology concept emphasizes the evidence-based transformation of edible natural materials into functional resources with defined bioactive components and health-promoting effects. Moreover, environmental or stress conditions can alter the content and structure of active metabolites in food-medicine homologous materials and thereby enhance their biological functionality (38, 39). Tea polyphenols, particularly catechins such as EGCG, exhibit potent antioxidant, anti-inflammatory, and anticancer properties by scavenging free radicals, regulating redox-related pathways (*Nrf2*, *NF–κB*, and *MAPK*), and improving gut microbiota balance and epithelial integrity. These bioactive compounds contribute to the prevention and management of chronic diseases, including cardiovascular, metabolic, and inflammatory disorders, underscoring tea’s role as a functional beverage for human health (40, 41).

Although *E. vexans* infection causes tea blister blight, which is typically viewed as detrimental to tea production, recent metabolomic and phytochemical studies have shown that the infection induces the accumulation of flavonoids, phenolic acids, alkaloids, and terpenoids, potentially enhancing the bioactivity of infected tea leaves (33). However, the biological and pharmacological properties of tea blister blight-infected leaves remain largely unexplored, particularly with respect to their protective effects against oxidative and inflammatory stress *in vivo*. This study aimed to evaluate the protective effects of tea blister blight aqueous extract (TBB) against CuSO_4_-induced oxidative and inflammatory stress in zebrafish embryos by assessing survival, hatching, morphology, oxidative stress biomarkers, and gene expression related to oxidative stress, inflammation, and apoptosis.

## 2 Materials and methods

### 2.1 Materials

Tea leaves showing typical symptoms of tea blister blight were harvested from Hongmin Tea Garden, Duyun City, Qiannan Buyi and Miao Autonomous Prefecture, Guizhou Province, China. Symptomatic leaves were selected based on visible diagnostic features, including translucent spots, raised blister-like lesions, blister depressions, and necrotic blister spots, following the symptom-stage criteria described in our previous study on *E. vexans*-infected tea leaves (33). No additional molecular confirmation, such as PCR or ITS sequencing, was performed. Copper (II) sulfate pentahydrate (CuSO_4_·5H_2_O), superoxide dismutase (SOD), malondialdehyde (MDA), catalase (CAT), bicinchoninic acid (BCA) protein concentration assay kits; a reverse transcription kit, a PCR reaction kit, PCR primers; 2-diphenyl-1-picrylhydrazyl (DPPH); phosphate-buffered saline (PBS); and TRIzol reagent were purchased from Sangon Biotech Co., Ltd. (Shanghai, China). Ethyl aminobenzoic acid ethyl ester methanesulfonate (MS-222), E3 embryo culture medium, and wild-type AB strain zebrafish (*D. rerio*) were obtained from Shanghai Feixi Biotechnology Co., Ltd. (Shanghai, China).

### 2.2 Preparation of tea blister blight aqueous extract and composition analysis

Tea leaves infected with tea blister blight disease were air-dried and subsequently oven-dried to remove residual moisture. The dried material was ground using a BFL-YB1500 Tea Grinder (Yongkang Xicheng Ouchuan Motor Factory, China) and passed through a 40-mesh sieve to obtain a fine powder. For each extraction, 5 g of the powder was mixed with 90 mL of water and extracted in an 85 °C DK-8D Three-Hole Electric Thermostatic Water Tank (Shanghai Yiheng Technology Co., Ltd., China) for 90 min. The resulting solution was filtered through a 0.22 μm PVDF membrane to remove insoluble residues and diluted with water to a final concentration of 50 mg/mL to obtain the tea blister blight (TBB) aqueous extract stock solution. The stock solution was prepared fresh before use and stored at 4 °C for no longer than 48 h to maintain stability of the active components. In addition, the dried tea blister blight sample was subjected to liquid chromatography–tandem mass spectrometry to analyze its metabolite profile. Briefly, the analysis was performed using a Shimadzu Nexera X2 UPLC system equipped with an Applied Biosystems 4,500 QTRAP mass spectrometer with an Agilent SB-C18 column (1.8 μm, 2.1 mm × 100 mm). The mobile phases consisted of water with 0.1% formic acid and acetonitrile with 0.1% formic acid. The flow rate was 0.35 mL/min, the column temperature was 40 °C, and the injection volume was 4 μL (33).

### 2.3 Zebrafish maintenance and embryo collection

Healthy wild-type AB strain zebrafish (*D. rerio*) were maintained in a recirculating water system at 28 °C under controlled conditions (pH 7.5 ± 0.3; conductivity 500 μS/cm; 14 h light/10 h dark photoperiod) using a KRG-250 Constant Temperature Illumination Incubator (Shanghai Qixin Scientific Instrument Co., Ltd., China). Brine shrimp nauplii were provided twice daily at 12:00 and 18:00. For spawning, sexually mature males and females were placed in spawning tanks at a 1:1 (2:2) ratio and separated by a transparent partition overnight. The following morning, after lights were turned on (08:00), the partition was removed to stimulate natural spawning. At 1 h post fertilization (hpf), embryos were collected, rinsed three times with E3 medium or system water, and transferred to E3 culture medium. Embryos were incubated at 28 °C until 4 hpf, after which morphologically normal embryos (uniform cleavage and normal development) were selected for subsequent experiments (19, 42). All zebrafish experiments were approved by the Animal Experimental Ethics Committee of Qiannan Normal University for Nationalities, College of Biological Science and Agriculture (Approval No. 2024005, approved on 5 March 2024).

### 2.4 Embryo toxicity test

The zebrafish embryos at the 4-cell to 128-cell stage were cultured in 24-well plates. The experiment was carried out in CRD with 13 treatments varying TBB concentration of 0, 0.2, 0.4, 0.8, 1.0, 2.0, 4.0, 8.0, 10.0, 20.0, 30.0, 40.0, and 50.0 mg/mL, three replications, each containing 30 embryos per replication.

The embryos were exposed for 48 h, during which the treatment medium was renewed every 24 h, and embryo mortality was recorded using the following equation (43):

$$\;\text{Mortality rate}\;(\%)=\frac{\text{The number of zebrafish embryos dead}}{\text{Total zebrafish embryos}}\times 100$$

The LC_50_ value of TBB was calculated by regression probability using SPSS software (*p* < 0.05).

### 2.5 Zebrafish embryo exposure experiment

The experiment was carried out in CRD with eight treatments and three replications (*n* = 3), with 60 embryos per replication. The E3 culture medium served as the blank control (CK). A zebrafish embryo toxicity model was established using 3 μM CuSO_4_ (PC). Three TBB-only treatments (0.3, 0.6, and 0.9 mg/mL) were used to evaluate the effects of TBB. These concentrations were selected based on the LC₅₀ determined in the acute toxicity test. Three combination treatments consisting of TBB and CuSO_4_ (0.3 mg/mL + 3 μM CuSO_4_, 0.6 mg/mL + 3 μM CuSO_4_, and 0.9 mg/mL + 3 μM CuSO_4_) were used to evaluate the protective effects against CuSO_4_-induced toxicity. A volume of 2 mL of the treatment solution was added to each well containing three fertilized embryos, which were incubated at 28.5 ± 0.5 °C under a 14-h light/10-h dark photoperiod for 144 h in a KRG-250 Constant Temperature and Illumination Incubator (Shanghai Qixin Scientific Instrument Co., Ltd., Shanghai, China). The treatment solution was renewed every 24 h (18, 44).

### 2.6 Developmental and morphological effects

The number of live, dead, and hatched zebrafish embryos was recorded every 24 h. The survival and hatching rates were calculated using the following equation:

$$\text{Survival rate}\;(\%)=\frac{\text{The number of zebrafish embryos alive}}{\text{Total zebrafish embryos}}\times 100$$

$$\text{Hatching rate}\;(\%)=\frac{\text{The number of zebrafish embryos hatched}}{\text{Total zebrafish embryos}}\times 100$$

At 120 hpf, zebrafish larvae were anesthetized with 0.02% MS-222 for 10 s and then immediately transferred to fresh E3 medium to terminate anesthesia. Morphological parameters, including body length, heart rate, eye diameter, and sinus venosus–bulbus arteriosus distance (SV–BA) distance, were measured using an MSD-VH1000 Ultra-depth 3D Stereo Microscope (Shanghai Qixin Scientific Instrument Co., Ltd., China) (45, 46).

### 2.7 Oxidative stress biochemical assays

Oxidative stress-related markers were evaluated following the methods of Li et al. (46) with minor modifications. At 120 hpf, 20 zebrafish larvae from each replicate were randomly selected for each treatment (*n* = 3), rinsed with phosphate-buffered saline (PBS), and homogenized using a tissue/cell protein extraction kit. The homogenates were centrifuged at 4000 × *g* for 15 min at 4 °C, and the resulting supernatants were collected for biochemical assays of oxidative stress-related markers. Superoxide dismutase (SOD) activity was determined at 460 nm, catalase (CAT) activity at 540 nm, and malondialdehyde (MDA) levels at 540 nm, following the manufacturer’s instructions for the respective commercial assay kits (Sangon Biotech Co., Ltd., Shanghai, China). Protein concentration in each sample was quantified using a bicinchoninic acid (BCA) protein assay kit, and all enzymatic activities were normalized to the corresponding protein concentration.

### 2.8 RNA extraction and gene expression analysis

A total of 20 zebrafish larvae were randomly selected from each replicate (*n* = 3), rapidly frozen in liquid nitrogen, and stored at −80 °C for subsequent analysis. RNA was extracted using TRIzol reagent (Sangon Biotech Co., Ltd., Shanghai, China) following homogenization, phase separation, and precipitation. The extracted RNA was dissolved in DEPC-treated water, and its purity was assessed by measuring the A260/A280 ratio with a nucleic acid–protein analyzer. RNA concentration was estimated at 260 nm, and 2 ng of total RNA was used for reverse transcription and cDNA synthesis with a MightyScript Plus First Strand cDNA Synthesis Master Mix Kit (Sangon Biotech Co., Ltd., China). Quantitative real-time PCR (qPCR) was performed to evaluate the relative expression of antioxidant-related genes (*Mn-sod, CAT, Gpx1a*), inflammatory mediators (*NF–κB, NLRP3, TNF-α, IL-1β, IL-10*), and apoptosis-related genes (*bax, Bcl-2*). *β-actin* was used as the internal reference gene. Gene-specific primers were synthesized by Sangon Biotech Co., Ltd. (Shanghai, China), and their sequences are listed in Table 1. Amplification was conducted using a SYBR Green PCR kit. The qPCR cycling conditions were as follows: initial denaturation at 95 °C for 30 s, followed by 40 cycles of denaturation at 95 °C for 10 s and annealing/extension at 60 °C for 30 s. A melting curve analysis was performed to verify the specificity of the amplified products. Relative gene expression levels were calculated using the 2^−ΔΔCt^ method (46, 47).

**Table 1:** Primer sequences used in real-time quantitative PCR (qPCR).

Gene	Function	Sequence Forward (F) and Reverse (R)
*β-actin*	Housekeeping	F: ATGGATGAGGAAATCGCTGCC R: CTCCCTGATGTCTGGGTCGTC
Glutathione peroxidase (*GPxla*)	Antioxidant	F: CACCCTCTTGTTTGCGTTCC R: TGTGAGTCTCAGCACACTTCCATC
Catalase (*CAT*)	Antioxidant	F: TCCGGACATGGTTTGGGAT R: CGATCCGCTTCTTCAACAGG
Manganese superoxide dismutase (*Mn-sod*)	Antioxidant	F: AGCGTGACTTTGGCTCATTT R: ATGAGACCTGTGGTCCCTTG
Tumor necrosis factor (*TNF-α*)	Proinflammatory cytokine	F: GCTGGATCTTCAAAGTCGGGTGTA R: TGTGAGTCTCAGCACACTTCCATC
NOD-like receptor family pyrin domain containing protein 3 (*NLRP3*)	Inflammasome activation	F: TTGTCTGGCTGTATGGTG R: TGATTGTCCTGGGTGATT
*IL-1β*	Inflammation	F: ATGGCAGAAGTACCTAAGCTC R: TTAGGAAGACACAAATTGCATGGTGAACTCAGT
*NF-kB*	Inflammation	F: ACAAGACGCAAGGAGCCCAG R: AACTGTCTCTTGCACAAAGGGCTCA
Interleukin −10 (*IL-10*)	Anti-inflammatory cytokine	F: CGGGATATGGTGAAATGCAAGA R: AGAGCAAATCAAGCTCCCCC
Bcl2-associated X (*bax*)	Apoptosis	F: GCGATACGGGCAGTGGCAATG R: GCGTTTATGGCTGGGGTCACAC
B-cell lymphoma-2 (*Bcl-2*)	Mitochondrial proapoptosis	F: TGGCGTCCCAGGTAGATAATATTGC R: CGCAGAGGCTGTCACTTCTGAGC

### 2.9 Data analysis

All experimental data were analyzed using one-way analysis of variance (ANOVA) with IBM SPSS Statistics 25.0. *Post hoc* comparisons among groups were performed using Duncan’s multiple range test. Statistical significance was set at *p* < 0.05, with different lowercase letters (a, b, c) denoting significant differences, while groups sharing the same letter were considered not to differ significantly. Quantitative data were expressed as the mean ± standard deviation (M ± SD). Linear trend analysis was performed for the CuSO_4_ and CuSO_4_ + TBB treatment groups, using TBB concentration as the independent variable and each biochemical or gene expression parameter as the dependent variable. The fitted linear trend lines and coefficients of determination (*R*^2^) were added to the figures to illustrate concentration-dependent protective effects. Graphical analyses and data visualization were performed using Origin 2024b (OriginLab, Northampton, MA, United States) and Microsoft Excel 2021.

## 3 Results

### 3.1 Metabolite analysis in tea blister blight aqueous extract

**Figure 1:**
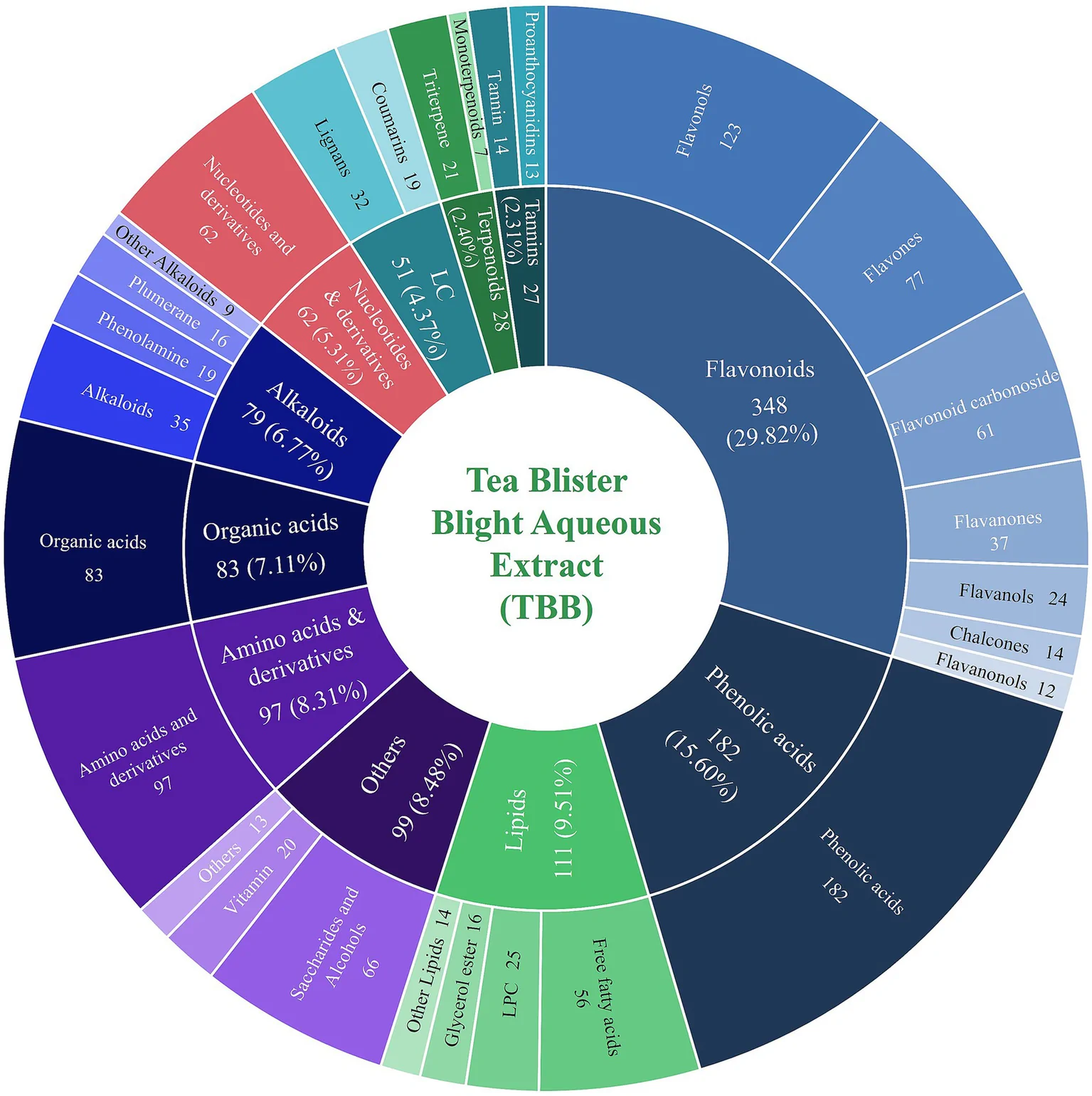
Classification of metabolite compounds in blister blight aqueous extract (TBB). Other alkaloids (4 pyridine alkaloids, 2 pyrrole alkaloids, 19 phenolamine, 1 tropan alkaloids, 1 quinoline alkaloids, 1 isoquinoline alkaloids); Other lipids (2 sphingolipids, 12 LPE).

Metabolite profiling of TBB identified a total of 1,167 compounds, which were categorized into 11 major chemical classes (Supplementary Table S1). The largest group was flavonoids (348 compounds, 29.82%), including flavonols (10.54%), flavone glycosides (5.23%), flavones (6.60%), flavanones (3.17%), flavanols (2.06%), chalcones (1.20%), and flavanonols (1.03%) subclasses. Phenolic acids accounted for the second-largest category (182 compounds, 15.60%), followed by amino acids and derivatives (97 compounds, 8.31%), and lipids (111 compounds, 9.51%). Alkaloids were also prominently represented (79 compounds, 6.77%), including plumerane alkaloids (1.37%), phenolamines (1.63%), and other alkaloid subclasses (0.77%). Organic acids constituted 83 compounds (7.11%), whereas nucleotides and derivatives accounted for 62 compounds (5.31%). Additional classes included lignans and coumarins (51 compounds, 4.37%), tannins (27 compounds, 2.31%), and terpenoids (28 compounds, 2.40%), primarily triterpenes (1.80%) and monoterpenoids (0.60%), and other metabolites (99 compounds, 8.48%), including saccharides and alcohols (5.66%), and vitamins (1.71%) (Figure 1).

### 3.2 Effects of tea blister blight aqueous extract on mortality rate, survival rate, and hatching rate of zebrafish embryos

**Figure 2:**
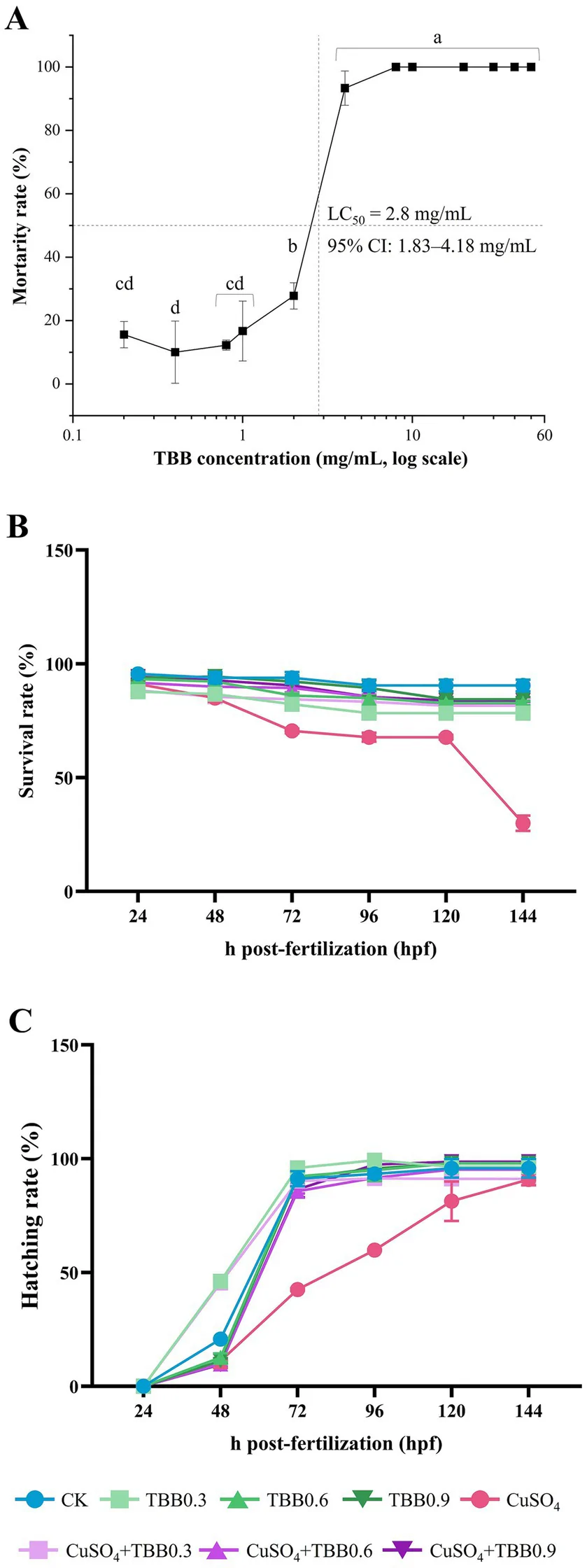
Effect of blister blight aqueous extract (TBB) treatments on **(A)** mortality rate, **(B)** survival rate, and **(C)** hatching rate of *zebrafish* embryos. CK (blank control), TBB0.3 (0.3 mg/mL TBB), TBB0.6 (0.6 mg/mL TBB), TBB0.9 (0.9 mg/mL TBB), CuSO_4_ (3 μM CuSO_4_), CuSO_4_ + TBB0.3 (3 μM CuSO_4_ + 0.3 mg/mL TBB), CuSO_4_ + TBB0.6 (3 μM CuSO_4_ + 0.6 mg/mL TBB), CuSO_4_ + TBB0.9 (3 μM CuSO_4_ + 0.9 mg/mL TBB). All data are expressed as mean ± SD (*n* = 3), and different lowercase letters represent statistically significant differences among the treatments (*p* < 0.05).

The mortality rate was 21.11% in the TBB control group (0 mg/mL). Exposure to low TBB concentrations of 0.2 to 1 mg/mL did not increase embryo mortality (10.00–16.67%), whereas TBB at 2 mg/mL significantly increased the mortality rate to 27.78%. At higher concentrations, TBB significantly increased embryo mortality, reaching 93.33% at 4 mg/mL and 100% at 8–50 mg/mL. The LC_50_ of TBB was calculated to be 2.8 mg/mL (95% confidence interval [CI], 1.83–4.18 mg/mL). Based on the ecotoxicological safety threshold principle, one-tenth of the LC_50_ (0.28 mg/mL) was considered the minimum effective concentration (Figure 2A). Accordingly, three concentrations (0.3, 0.6, and 0.9 mg/mL) were selected for the zebrafish embryo exposure experiment.

The survival rate of zebrafish embryos in the control group (CK) remained stable at approximately 90.56% up to 144 hpf. In contrast, the positive control group exposed to CuSO_4_ (PC) showed a significant decline in survival, decreasing to 30.00% at 144 hpf (*p* < 0.05). Treatment with TBB alone (0.3–0.9 mg/mL) resulted in survival rates ranging from 78.3 to 84.4% at 144 hpf, which were slightly lower than those in the CK group but significantly higher than those in the PC (*p* < 0.05). Moreover, the combined treatment groups (CuSO_4_ + TBB) demonstrated survival rates of 81.67–83.89% at 144 hpf, 12.74–15.81% compared with the PC group (Figure 2B).

The hatching rate reached 20.71% at 48 hpf and exceeded 95.78% by 120 hpf in the CK group. In the PC group, hatching was significantly delayed compared with that in the CK, with only 11.13% of embryos hatched at 48 hpf and 90.88% by 144 hpf. Embryos treated with TBB at 0.3 mg/mL exhibited a higher hatching rate (46.18%) at 48 hpf, comparable to the CK group, whereas hatching at the other TBB concentrations was delayed and comparable to that in the PC group. However, all TBB-treated groups reached 96.47 to 98.03% by 144 hpf. Co-treatment with CuSO_4_ and TBB at 0.3 mg/mL significantly increased the hatching rate to 45.40%, whereas the increases at the other concentrations were not significant compared with the PC group at 48 hpf. However, by 144 hpf, the hatching rates in the CuSO_4_ + TBB groups at 0.6–0.9 mg/mL were 95.27–98.70%, representing increases of 46.12–51.38% relative to the PC group (*p* < 0.05) (Figure 2C).

### 3.3 Effects of tea blister blight aqueous extract on morphological zebrafish

**Figure 3:**
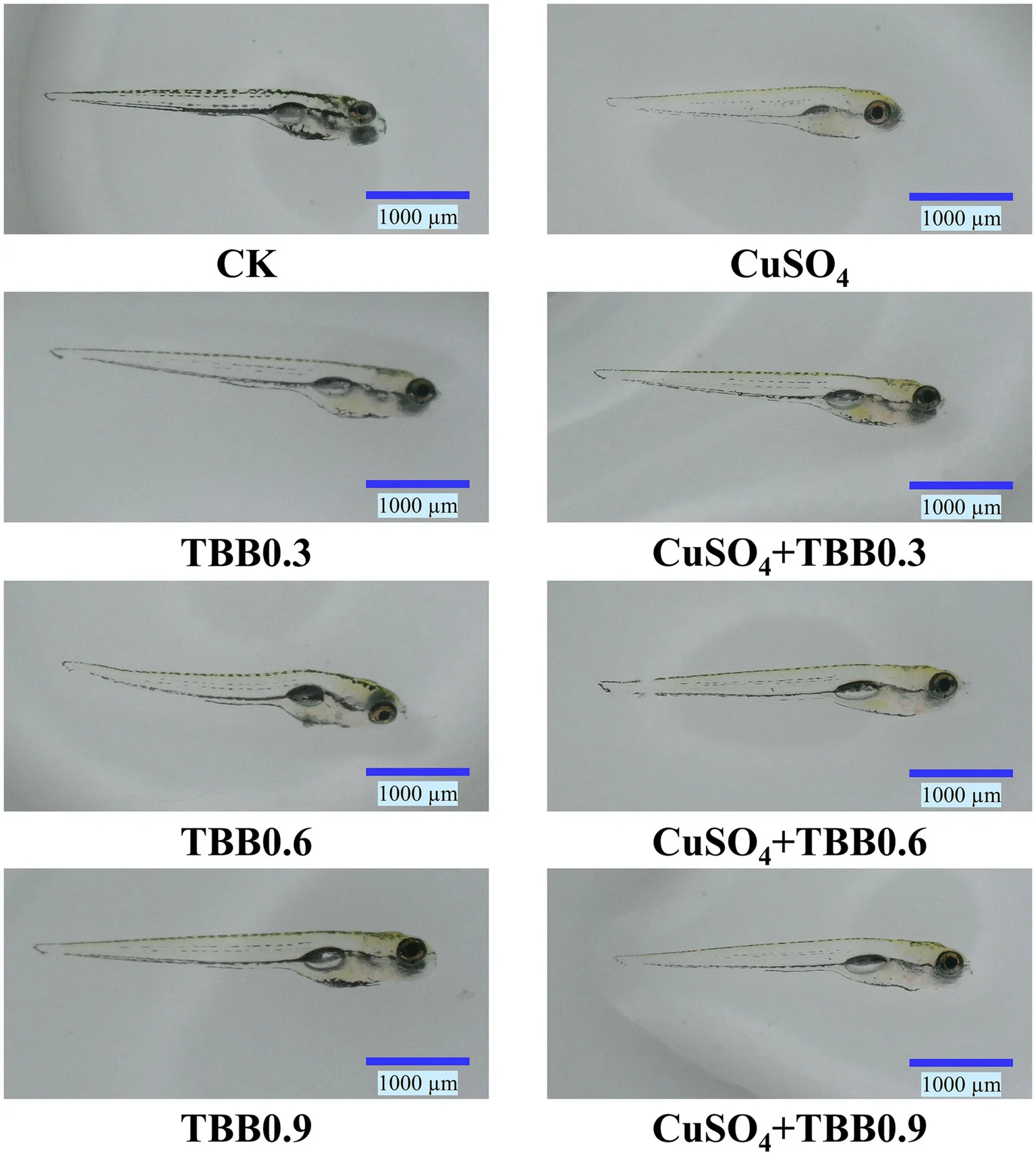
Effect of blister blight aqueous extract (TBB) treatments on morphological *zebrafish*. CK (blank control), TBB0.3 (0.3 mg/mL TBB), TBB0.6 (0.6 mg/mL TBB), TBB0.9 (0.9 mg/mL TBB), CuSO_4_ (3 μM CuSO_4_), CuSO_4_ + TBB0.3 (3 μM CuSO_4_ + 0.3 mg/mL TBB), CuSO_4_ + TBB0.6 (3 μM CuSO_4_ + 0.6 mg/mL TBB), CuSO_4_ + TBB0.9 (3 μM CuSO_4_ + 0.9 mg/mL TBB).

Morphological parameters of zebrafish larvae at 120 hpf are presented in Table 2 and Figure 3. In the control group (CK), larvae exhibited normal heart rate (124 ± 1.5 beats/min), SV–BA distance (294 ± 3.7 μm), eye diameter (301 ± 1.8 μm), and body length (4,246 ± 11.5 μm). Exposure to CuSO_4_ (PC) significantly impaired larval development, as evidenced by reductions in heart rate (104 ± 1.7 beats/min), SV–BA distance (282 ± 3.1 μm), eye diameter (250 ± 1.9 μm), and body length (4,168 ± 12.5 μm) compared with the CK group (*p* < 0.05). Treatment with TBB alone at 0.3–0.9 mg/mL did not adversely affect larval morphology, as body length, eye diameter, SV–BA distance, and heart rate did not differ significantly from those in the CK group (*p* > 0.05). Moreover, co-treatment with TBB and CuSO_4_ significantly attenuated the CuSO₄-induced morphological abnormalities, although the measured parameters remained lower than those in the CK group. In the TBB co-treated groups, heart rate and SV-BA distance increased by 15.38–16.35% and 10.40–14.80%, respectively, compared with the PC group. Only TBB at 0.9 mg/mL significantly increased body length by 1.37% relative to the PC group.

**Table 2:** Effect of tea blister blight aqueous extract (TBB) treatments on morphological parameters of *zebrafish* larvae.

Treatment^a^	Heart rate (beat/min)	SV-BA (μm)	Eye length (μm)	Body length (μm)
CK	124 ± 1.5ab	294 ± 3.7abc	301 ± 1.8b	4,246 ± 11.5a
TBB0.3	125 ± 2.2a	298 ± 4.7ab	304 ± 0.9a	4,251 ± 29.7a
TBB0.6	126 ± 1.2a	297 ± 5ab	305 ± 1.5a	4,252 ± 18.6a
TBB0.9	126 ± 1.7a	299 ± 7.9a	305 ± 1.4a	4,258 ± 19.2a
CuSO_4_	104 ± 1.7d	282 ± 3.1c	250 ± 1.9d	4,168 ± 12.5c
CuSO_4_ + TBB0.3	120 ± 1.6c	287 ± 3.8bc	276 ± 1.9c	4,188 ± 27.7bc
CuSO_4_ + TBB0.6	120 ± 2.6c	289 ± 5.4bc	273 ± 2c	4,204 ± 29.5bc
CuSO_4_ + TBB0.9	121 ± 2bc	289 ± 3.2bc	287 ± 2.3bc	4,225 ± 28.3ab
F-test	*	*	*	*

a CK (blank control), TBB0.3 (0.3 mg/mL TBB), TBB0.6 (0.6 mg/mL TBB), TBB0.9 (0.9 mg/mL TBB), CuSO_4_ (3 μM CuSO_4_), CuSO_4_ + TBB0.3 (3 μM CuSO_4_ + 0.3 mg/mL TBB), CuSO_4_ + TBB0.6 (3 μM CuSO_4_ + 0.6 mg/mL TBB), CuSO_4_ + TBB0.9 (3 μM CuSO_4_ + 0.9 mg/mL TBB).

All data are expressed as mean ± SD (*n* = 3), and different lowercase letters represent statistically significant differences among the treatments at 0.01 < *p* ≤ 0.05 (*) and *p* ≤ 0.01 (**).

### 3.4 Effects of tea blister blight aqueous extract on oxidative stress-related biochemical parameters

**Figure 4:**
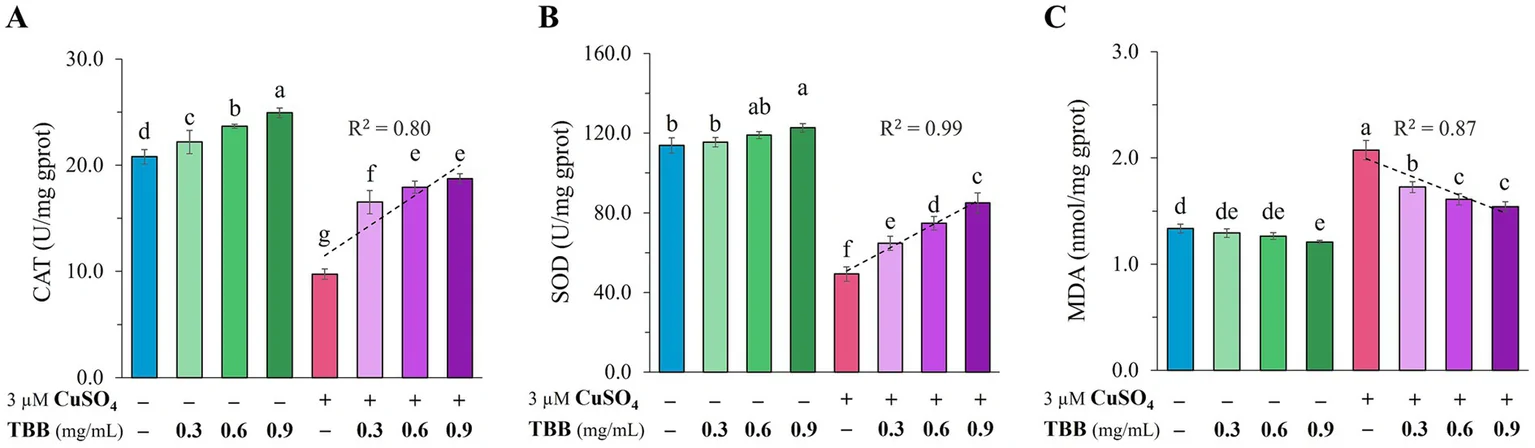
Effect of blister blight aqueous extract (TBB) treatments on oxidative stress-related biochemical parameters including **(A)** CAT, **(B)** SOD, and **(C)** MDA in *zebrafish* (*Danio rerio*) larvae. All data are expressed as mean ± SD (*n* = 3), and different lowercase letters represent statistically significant differences among the treatments (*p* < 0.05). Dashed black lines were fitted linear trends for the CuSO_4_ and CuSO_4_ + TBB co-treatment groups; *R*^2^ values were shown in each graph.

The activities of antioxidant enzymes and the levels of lipid peroxidation products in zebrafish larvae are presented in Figure 4. In the control group (CK), CAT and SOD activities were maintained at normal levels, whereas MDA levels remained low. Exposure to CuSO_4_ (PC) significantly suppressed CAT and SOD activities by 53.18 and 56.74%, respectively, compared with the CK group (*p* < 0.05). The PC significantly increased MDA accumulation by 55.17%, indicating lipid peroxidation and oxidative damage. Treatment with TBB enhanced antioxidant activity in zebrafish. All TBB treatments significantly increased CAT activity by 6.68–19.85%, whereas only TBB at 0.9 mg/mL significantly increased SOD activity by 7.71% and decreased MDA levels by 9.63% compared with the CK group (*p* < 0.05). Moreover, co-treatment with CuSO_4_ and TBB effectively mitigated CuSO_4_-induced oxidative stress. CAT and SOD activities were significantly increased by 69.66–92.37% and 31.29–72.35%, respectively, compared with the PC group (*p* < 0.05). In addition, MDA levels were reduced by 16.74–25.61% compared with the PC group. Linear trend analysis further supported concentration-dependent increases in CAT and SOD activities (*R*^2^ = 0.80 and 0.99, respectively) and a concentration-dependent reduction in MDA levels (*R*^2^ = 0.87) in the CuSO_4_ + TBB groups. However, CAT and SOD activities and MDA levels in the CuSO₄ + TBB groups (0.3–0.9 mg/mL) were not fully restored to the levels observed in the CK group.

### 3.5 Gene expression analysis

#### 3.5.1 Oxidative stress-related genes

**Figure 5:**
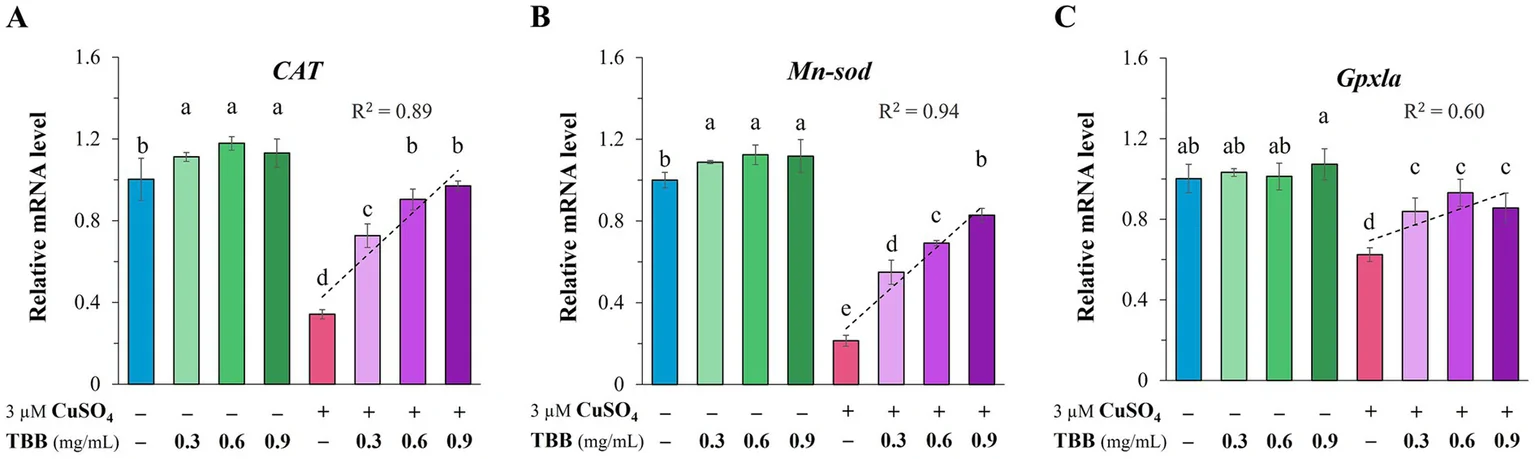
Effect of blister blight aqueous extract (TBB) treatments on mRNA expression of oxidative stress-related genes including **(A)** *CAT*, **(B)** *Mn-*SOD and **(C)** *GPx*1*a* in *zebrafish* (*Danio rerio*) larvae. All data are expressed as mean ± SD (*n* = 3), and different lowercase letters represent statistically significant differences among the treatments (*p* < 0.05). Dashed black lines were fitted linear trends for the CuSO_4_ and CuSO_4_ + TBB co-treatment groups; *R*^2^ values were shown in each graph.

The relative mRNA expression of antioxidant-related genes (*CAT*, *Mn-sod*, and *Gpx1a* referring to zebrafish gene symbols) is presented in Figure 5. In the control group (CK), the basal expression of these genes was maintained at normal levels. Exposure to CuSO_4_ (PC) significantly downregulated the *CAT*, *Mn-sod*, and *Gpx1a* expression by 2.93-, 4.66-, and 1.61-fold, respectively, compared with the CK group (*p* < 0.05), indicating damage or impairment of the antioxidant defense system at the transcriptional level. Treatment with TBB only (0.3–0.9 mg/mL) upregulated the expression of *CAT* and *Mn-sod* by 1.11- to 1.17-fold and 1.09-fold to 1.12-fold, respectively, whereas no significant effect was observed for *Gpx1a*. Co-treatment with CuSO_4_ and TBB effectively reversed CuSO_4_-induced gene suppression. The expression of *CAT*, *Mn-sod*, and *Gpx1a* was significantly upregulated by 2.13- to 2.84-fold, 2.56- to 3.86-fold, and 1.34- to 1.49-fold, respectively, compared with CuSO_4_ exposure (the PC group) (*p* < 0.05). Linear trend analysis supported concentration-dependent recovery of *CAT* and *Mn-sod* expression (*R*^2^ = 0.89 and 0.94, respectively), whereas *Gpx1a* showed only a partial recovery trend (*R*^2^ = 0.60). Specifically, TBB at 0.9 mg/mL restored *CAT* and *Mn-sod* expression to levels comparable to those in the CK group.

#### 3.5.2 Inflammation-related genes

**Figure 6:**
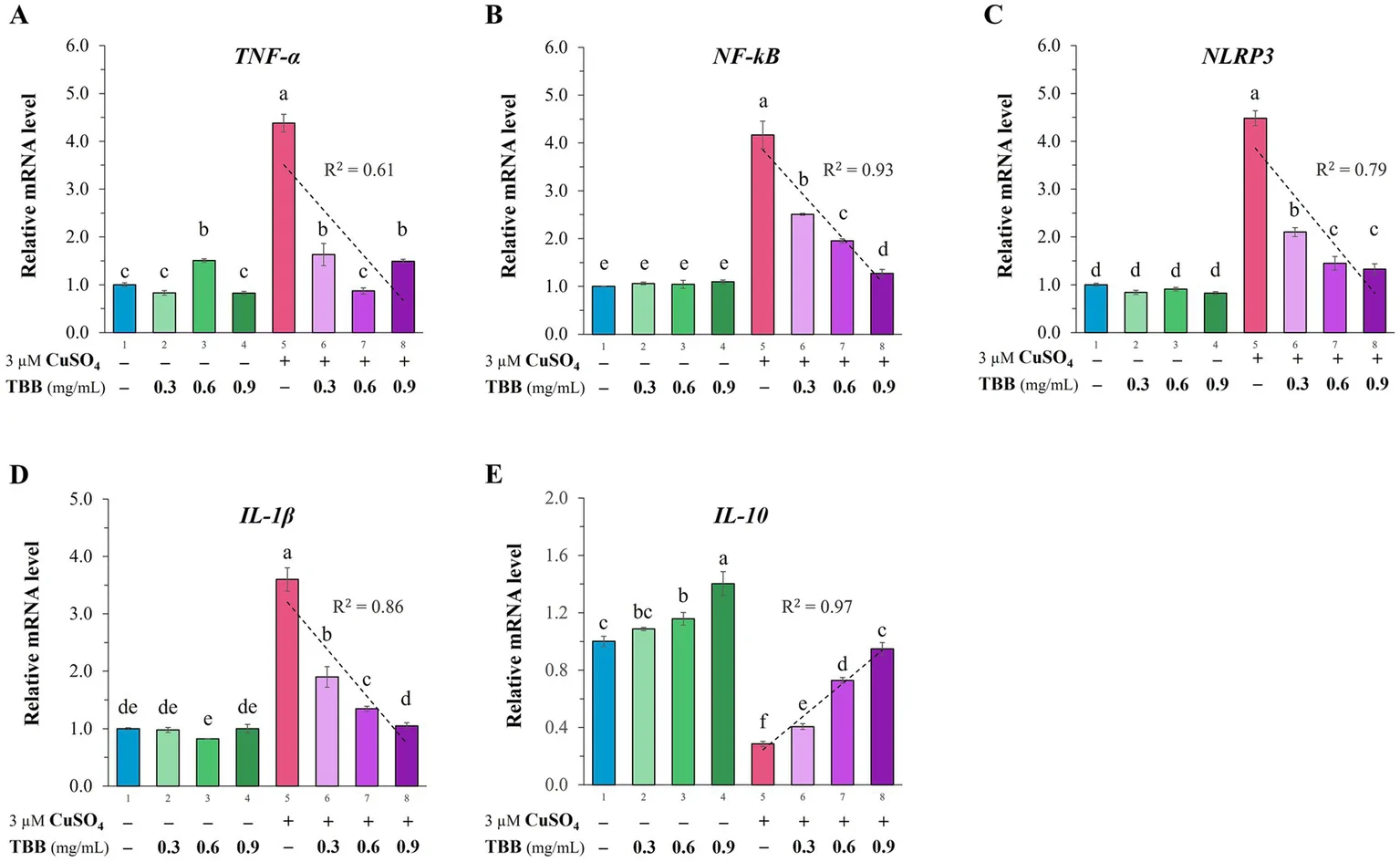
Effect of blister blight aqueous extract (TBB) treatments on mRNA expression of inflammation-related genes, including **(A)** *TNF-α*, **(B)** *NF–κB,* **(C)** *NLRP3*, **(D)** *IL-1β*, and **(E)** *IL-10* in *zebrafish* (*Danio rerio*) larvae. All data are expressed as mean ± SD (*n* = 3), and different lowercase letters represent statistically significant differences among the treatments (*p* < 0.05). Dashed black lines were fitted linear trends for the CuSO_4_ and CuSO_4_ + TBB co-treatment groups; *R*^2^ values were shown in each graph.

The expression profiles of pro- and anti-inflammatory genes in zebrafish larvae are presented in Figure 6. CuSO_4_ exposure (PC) significantly upregulated the mRNA expression of proinflammatory genes, including *TNF-α* (4.48-fold), *NF–κB* (4.17-fold), *NLRP3* (4.48-fold), and *IL-1β* (3.6-fold) compared with the control group (CK) (*p* < 0.05), indicating activation of inflammatory pathways. In parallel, the anti-inflammatory cytokine *IL-10* was significantly downregulated by 3.5-fold in the PC group compared with the CK group (*p* < 0.05). Treatment with TBB only (0.3–0.9 mg/mL) did not induce proinflammatory gene expression, except that TBB at 0.6 mg/mL significantly upregulated *TNF-α* by 1.5-fold compared with the CK group. TBB at 0.6–0.9 mg/mL significantly upregulated anti-inflammatory *IL-10* expression in a dose-dependent manner by 1.16- to 1.4-fold compared with the CK group (*p* < 0.05). Co-treatment with CuSO_4_ and TBB effectively suppressed the CuSO_4_-induced overexpression of proinflammatory genes, including *TNF-α* (2.68- to 5.04-fold), *NF–κB* (1.66- to 3.27-fold), *NLRP3* (2.13- to 3.38-fold), and *IL-1β* (1.89- to 3.43-fold) compared to PC (*p* < 0.05). Specifically, the TBB (0.6 mg/mL) and TBB (0.9 mg/mL) could restore *TNF-α* and *IL-1β* expression similar to CK, respectively. Linear trend analysis supported concentration-related suppression of *NF–κB*, *NLRP3*, and *IL-1β* (*R*^2^ = 0.93, 0.79, and 0.86, respectively), while *TNF-α* showed only a partial linear trend (*R*^2^ = 0.61). In addition, co-treatment with CuSO_4_ and TBB also significantly upregulated *IL-10* expression by 1.42- to 3.32-fold compared with PC (*p* < 0.05), with a strong concentration-related trend (*R*^2^ = 0.97). Specifically, TBB (0.9 mg/mL) could restore the *IL-10* level similar to CK.

#### 3.5.3 Apoptosis-related genes

**Figure 7:**
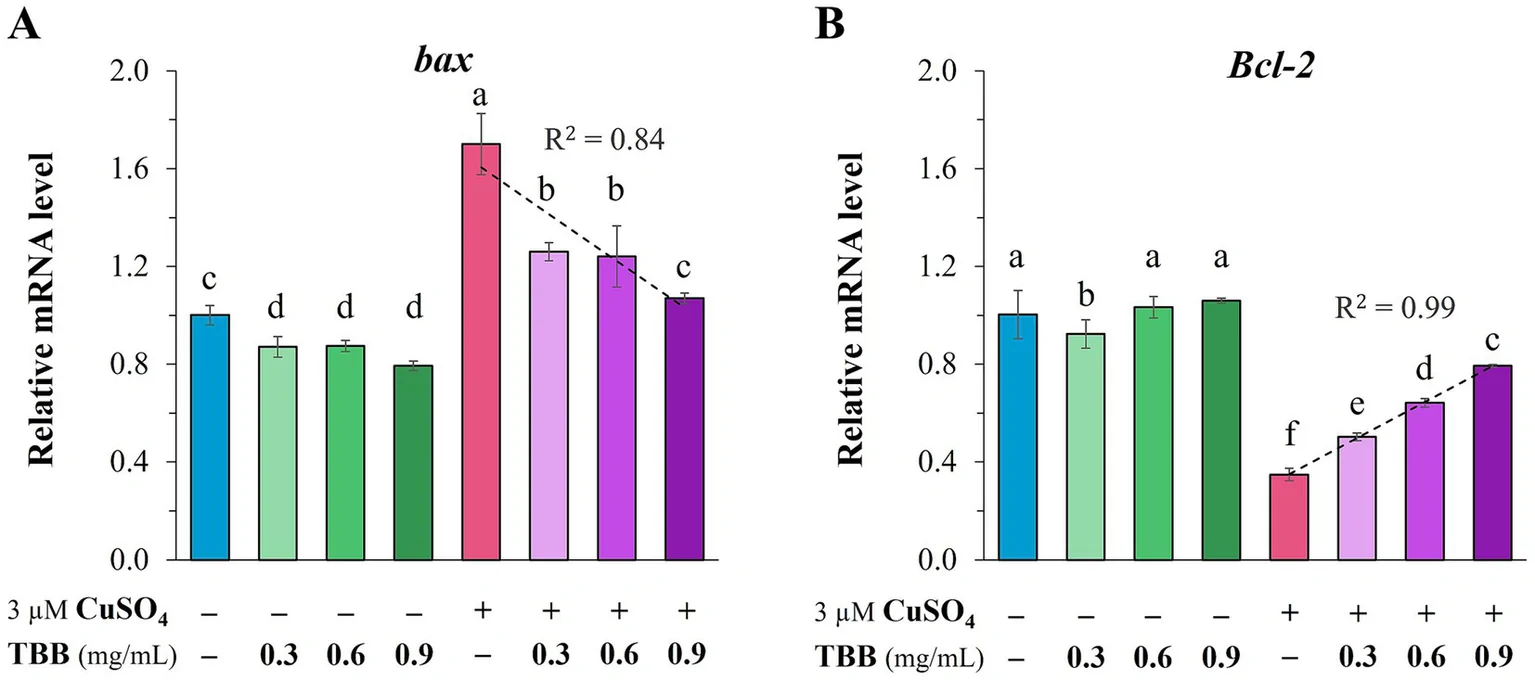
Effect of tea blister blight aqueous extract (TBB) treatments on mRNA expression of apoptosis-related genes including **(A)** *bax and* **(B)** *Bcl-2* in *zebrafish* (*Danio rerio*) larvae. All data are expressed as mean ± SD (*n* = 3), and different lowercase letters represent statistically significant differences among the treatments (*p* < 0.05). Dashed black lines were fitted linear trends for the CuSO_4_ and CuSO_4_ + TBB co-treatment groups; *R*^2^ values were shown in each graph.

The expression levels of apoptosis-related genes (*bax* and *Bcl-2*) in zebrafish larvae are presented in Figure 7. CuSO_4_ exposure (PC) significantly upregulated the pro-apoptotic gene *bax* by 1.7-fold (*p* < 0.05) and significantly downregulated the anti-apoptotic gene *Bcl-2* by 2.88-fold (*p* < 0.05), indicating activation of apoptotic signaling pathways. Treatment with TBB only balanced apoptosis-related gene expression. In this, *bax* levels were significantly reduced relative to CK by 1.14- to 1.26-fold, while *Bcl-2* expression was similar to CK except for TBB (0.3 mg/mL). Co-treatment with CuSO_4_ and TBB mitigated CuSO_4_-induced apoptosis. In the CuSO_4_ + TBB groups, *bax* expression was significantly downregulated compared with PC by 1.35- to 1.59-fold, while *Bcl-2* expression was restored by 1.44- to 2.28-fold compared with PC (*p* < 0.05). Linear trend analysis indicated concentration-related *bax* downregulation (*R*^2^ = 0.84) and strong *Bcl-2* restoration (*R*^2^ = 0.99) in the CuSO_4_ + TBB co-treatment groups. Specifically, TBB (0.9 mg/mL) could restore *bax* expression similar to CK.

## 4 Discussion

Tea blister blight is caused by *E. vexans*, which has traditionally been regarded as a destructive foliar disease that compromises tea yield and sensory quality (28, 29). However, pathogen infection can reprogram host plant secondary metabolism as part of an inducible defense response, leading to the accumulation of bioactive compounds with enhanced antioxidant and antimicrobial potential (36, 37). In our previous research, metabolomic profiling demonstrated that TBB possesses a chemically enriched and biologically distinct profile compared with healthy tea, providing a mechanistic basis for its observed protective effects *in vivo* (33). In the present research, untargeted metabolite analysis revealed that TBB contained 1,167 metabolites distributed across 11 major chemical classes, with flavonoids representing the most abundant group (30%), followed by phenolic acids, alkaloids, lipids, and organic acids. The flavonoid fraction was dominated by flavonols, flavones, and flavone glycosides, which are widely recognized for their strong redox-modulating, anti-inflammatory, and cytoprotective properties (13). Blister blight infection has been shown to induce isomerization and gallate esterification of catechins, alter proanthocyanidin configurations, and enhance the production of flavonols and phenolic acids that exhibit greater chemical stability and biological activity under stress conditions (30–32). These changes distinguish infected tea leaves from healthy tea not only in sensory attributes but also in functional bioactivity. The enrichment of flavonoid subclasses and phenolic acids observed in TBB aligns with prior metabolomic studies demonstrating that pathogen-stressed tea leaves can exhibit enhanced antioxidant capacity and altered biological functionality (33). In this context, tea blister blight should not be viewed solely as an agronomic liability but also as a source of a stress-primed phytochemical matrix with potential functional value when appropriately utilized.

The zebrafish embryo is a well-established vertebrate model for studying oxidative stress-related toxicity because of its high genetic similarity to humans and optical transparency during early development, which enables direct assessment of developmental and cellular responses *in vivo* (48, 49). Conserved redox, inflammatory, and apoptotic pathways further support its use for high-throughput screening of antioxidant and anti-inflammatory bioactive compounds (50). CuSO_4_, commonly used as an algaecide and fungicide, induces oxidative stress in zebrafish by promoting ROS generation and activating proinflammatory signaling pathways, including *TNF-α-* and *COX-2-*mediated pathways, making it a reliable *in vivo* model for evaluating protective interventions (19, 20). To evaluate the protective efficacy of TBB, it was essential to first confirm that CuSO_4_ exposure produced a robust and reproducible oxidative-inflammatory injury phenotype in zebrafish embryos. In the present study, exposure to 3 μM CuSO_4_ successfully induced a spectrum of developmental, biochemical, and molecular alterations characteristic of oxidative stress-driven toxicity, thereby validating the suitability of this model for mechanistic assessment. At the tested concentrations (0.3–0.9 mg/mL), TBB did not induce developmental toxicity in zebrafish embryos, as it did not cause negative effects on survival and hatching rates, morphological parameters including body length, eye size, SV–BA distance, and heart rate. TBB exhibited a pronounced protective effect against CuSO_4_-induced oxidative and inflammatory injury, significantly mitigating developmental impairment and physiological dysfunction. These protective effects were associated with the phytochemical composition of TBB, which included bioactive flavonoids and phenolic acids such as quercetin, kaempferol, and their glycosides (rutin and vitexin), chlorogenic acid and its isomers (5-caffeoylquinic acid and neochlorogenic acid), as well as other constituents including epigallocatechin gallate, sesamin, and related alkaloids and terpenoids. Flavonoids are important plant secondary metabolites with antioxidant, anti-inflammatory, antitumor, antiviral, and antibacterial activities, and their accumulation and antioxidant capacity are markedly influenced by environmental and biotic stress conditions (51).

Oxidative stress represents a central mechanism underlying CuSO_4_-induced toxicity, primarily through excessive ROS generation, lipid peroxidation, and impairment of endogenous antioxidant systems (1, 2). In the present study, CuSO_4_ exposure significantly suppressed SOD and CAT activities, increased MDA levels, and downregulated antioxidant-related genes (*Mn-sod, CAT*, and *Gpx1a*), indicating severe disruption of redox homeostasis. Co-treatment with TBB significantly reversed these alterations in a concentration-dependent manner, demonstrating effective restoration of antioxidant defenses at both biochemical and transcriptional levels.

Chlorogenic acid and its isomers, including 5-caffeoylquinic acid and neochlorogenic acid, exhibit strong radical-scavenging capacity and effectively reduce lipid peroxidation while enhancing SOD, CAT, and glutathione-dependent antioxidant systems in zebrafish exposed to heavy metals or metabolic stressors (52–55). *In silico* and molecular dynamics studies have demonstrated that caffeoylquinic acid derivatives inhibit xanthine oxidase, thereby limiting ROS generation at its enzymatic source (56). In addition, catechin has been shown to restore glutathione homeostasis and suppress oxidative stress-related genes in chlorpyrifos-exposed zebrafish (57). On the contrary, succinic acid—one of the major compounds present in TBB—enhanced CAT activity and *Nrf2*-regulated antioxidant gene expression in feeding trials involving Nile tilapia and large yellow croaker (58, 59).

Flavonols such as quercetin, kaempferol, and their derivatives further contribute to redox regulation by preserving mitochondrial function, stabilizing antioxidant enzymes, and modulating redox-sensitive transcriptional programs. Quercetin, quercetin nanocrystals, and kaempferol derivatives protect zebrafish, rat, and human cells from CuSO_4_-, H_2_O_2_-, and NaIO_3_-induced oxidative injury by restoring *Mn-SOD* activity, maintaining mitochondrial membrane potential, and limiting ROS-driven damage (60–62). Rutin and vitexin also enhance antioxidant capacity through activation of *Nrf2*-associated pathways and reduction of oxidative biomarkers in zebrafish exposed to phenylthiourea or acrylamide models (35, 63).

Oxidative stress and inflammation are tightly interconnected in CuSO_4_-induced toxicity, where ROS overproduction activates *NF–κB* signaling and inflammasome pathways, resulting in excessive pro-inflammatory cytokine release (6, 7). In the present study, CuSO_4_ exposure significantly upregulated *TNF-α*, *NF–κB*, *NLRP3*, and *IL-1β*, while suppressing the anti-inflammatory cytokine *IL-10*, indicating a pronounced pro-inflammatory state. Co-treatment with TBB effectively attenuated proinflammatory gene expression and restored *IL-10* expression toward physiological values, demonstrating a balanced immunomodulatory response. Epigallocatechin gallate (EGCG) suppressed *NF–κB* and *MAPK* signaling and reduced proinflammatory cytokine production in zebrafish and human cell models under oxidative stress (64). Rutin and rutin hydrate significantly inhibited *NF–κB*-dependent transcription of inflammatory-related genes (*IL6, TNF-α, IL10,* and *IRF2A*), thereby reducing neutrophil and macrophage recruitment, and attenuated neuroinflammation in zebrafish exposed to phenylthiourea or LPS (35, 44, 65). Kaempferol glycosides and chlorogenic acid derivatives similarly suppressed inflammatory mediators while activating *Nrf2/Keap1* signaling, which indicated redox immune regulation (54, 66). Interestingly, several TBB-related compounds exhibited dual regulation of inflammation by suppressing excessive proinflammatory signaling while enhancing anti-inflammatory mediators. Sesamin and optimal dietary leucine increased *IL-10* and *TGF-β* expression while reducing *TNF-α* and *IL-1β*, thereby promoting inflammatory resolution rather than broad immunosuppression (67–70). Moreover, salidroside exhibited dose-dependent immunomodulatory effects by enhancing host defense through *TNF-α-*mediated mechanisms without exacerbating inflammatory damage (71). Neohesperidin dihydrochalcone reduced pro-inflammatory cytokines (*TNF-α* and *IL-6*) and activated *PI3K/AKT*-mediated insulin signaling, thereby alleviating oxidative stress-associated inflammatory dysfunction in diabetic models (72).

Apoptosis is a critical downstream consequence of sustained oxidative stress-induced inflammation, particularly through mitochondrial dysfunction and disruption of the *Bcl-2/Bax* regulatory axis (8). In this study, CuSO_4_ exposure significantly increased the proapoptotic gene *bax* while downregulating the antiapoptotic gene *Bcl-2* expression, indicating activation of mitochondrial apoptotic signaling. TBB co-treatment significantly reversed these changes in a concentration-dependent manner, restoring the *Bcl-2/Bax* balance and suggesting suppression of apoptosis at the transcriptional level. Quercetin, kaempferol, vitexin, and their derivatives inhibit mitochondrial apoptosis by preserving mitochondrial membrane potential, suppressing caspase-3 activation, and restoring *Bcl-2/Bax* equilibrium in zebrafish and mammalian models exposed to oxidative stressors, including copper ions and hydrogen peroxide (60, 61, 73, 74). Catechin has also been shown to reduce apoptosis by downregulating *bax* and oxidative stress genes in zebrafish (57). Chlorogenic acid and neochlorogenic acid contribute to apoptosis prevention indirectly through activation of *Nrf2*-dependent antioxidant pathways, thereby limiting ROS-driven mitochondrial damage and secondary apoptotic signaling (54, 55). Conversely, compounds with known pro-oxidant effects, such as elaidic acid and excessive leucine exposure, induce oxidative stress, inflammation, and developmental toxicity, underscoring the specificity of protective mechanisms associated with TBB constituents (75, 76). While tea blister blight remains undesirable for premium tea production, infected leaves are typically discarded or undervalued despite their altered metabolite composition. The present study extends this paradigm by providing *in vivo* evidence that tea blister blight-infected leaves constitute a metabolically enhanced substrate with functional potential, rather than merely a degraded agricultural byproduct.

## 5 Conclusion

Tea blister blight aqueous extract (TBB) exhibited multilevel protective effects against copper sulfate-induced oxidative stress, inflammation, and apoptosis in zebrafish embryos. TBB significantly improved survival and hatching rates, reduced developmental and morphological impairments, decreased MDA accumulation, and enhanced antioxidant defenses through increased SOD and CAT activities. At the transcriptional level, TBB upregulated antioxidant-related genes (*Mn-sod, CAT,* and *Gpx1a*), suppressed proinflammatory mediators (*TNF-α, NF–κB, NLRP3, and IL-1β*), and the proapoptotic gene *bax*, while promoting anti-inflammatory (*IL-10*) and anti-apoptotic (*Bcl-2*) expression. These findings suggest that tea blister blight-infected leaves represent a bioactive-enriched resource with potential relevance for food–medicine homology applications. However, this study has several limitations. TBB was evaluated as a crude aqueous extract, and the specific active compounds responsible for the observed protective effects remain to be identified. In addition, the findings were obtained using a zebrafish embryo model and therefore cannot be directly extrapolated to humans. Future research should isolate and characterize the major bioactive compounds in TBB, clarify their molecular mechanisms of action, and evaluate the safety and efficacy of TBB or its active compounds in mammalian models before considering nutritional or functional applications.

## Data Availability

The original contributions presented in the study are included in the article/Supplementary material, further inquiries can be directed to the corresponding authors.
